# Competence committees decision-making; an interplay of data, group orientation, and intangible impressions

**DOI:** 10.1186/s12909-023-04693-4

**Published:** 2023-10-10

**Authors:** Colleen Curtis, Aliya Kassam, Jason Lord, Lara J. Cooke

**Affiliations:** 1 Department of Pediatrics, University of Calgary, 28 Oki Dr. NW, Calgary, AB T3B 6A8 Canada; 2 Department of Community Health Sciences, University of Calgary, Calgary, Canada; 3 Departments of Emergency Medicine and Critical Care Medicine, University of Calgary, Calgary, Canada; 4 Department of Clinical Neurosciences, University of Calgary, Calgary, Canada

**Keywords:** Competency-based medical education, Competence committees, Small group decisions

## Abstract

**Background:**

The implementation of competency-based medical education and utilization of competence committees (CC) represents a paradigm shift in residency education. This qualitative study aimed to explore the methods used by two operational CC and their members to make decisions about progression and competence of their residents.

**Methods:**

An instrumental case study methodology was used to study the CC of two postgraduate training programs. Transcripts from observed CC meetings, member interviews, and guiding documents were analyzed using a constructivist grounded theory approach to reveal themes explaining the decision-making process.

**Results:**

Our study found that the CC followed a process that began within a social decision schema model and evolved to a discussion that invoked social influence theory, shared mental models, and social judgment scheme to clarify the points of contention. We identified that the CC decision-making was at risk of bias, primarily influenced by the group composition, the group orientation and individual members’ mindset, as well as their personal experiences with the trainees.

**Conclusions:**

Increased awareness of the sources of bias in CC functioning and familiarity with the CC role in competency-based medical education would enable committees to provide valuable feedback to all trainees regardless of their trajectory.

**Supplementary Information:**

The online version contains supplementary material available at 10.1186/s12909-023-04693-4.

## Background

The implementation of competency-based medical education (CBME) represents a paradigm shift in postgraduate medical education to an outcomes-based curriculum [1]. In CBME, the determination of competence is intended to occur based on each trainee’s demonstration of the knowledge, skills and attitudes required for independent practice [2]. A purposively-designed program of assessment is required to understand the trainees’ trajectories, including multiple sources of assessment over time [3]. This should include a competence committee (CC), which makes high stakes recommendations about resident progression and promotion [3, 4]. In Canada, guidelines for CC have been published by the Royal College of Physicians and Surgeons of Canada (RCPSC) however each program is responsible for establishing its specific structure and goals in their CC Terms of Reference [5, 6]. Implementation of CBME in Canadian specialty residency training has been phased in, with the first programs starting the new framework of training and assessment in 2017. In this framework, residents are progressed through stages of training based on assessment of competence in performing key tasks, termed entrustable professional activities (EPA), as well as completion of in-training examinations and other required elements of training [7]. The EPA are observed and assessed in the moment by preceptors using a workplace-based assessment tool (WBA) with a 5-point scale (4–5 represent EPA-specific competence). While supervising physicians can offer to complete a WBA, the resident is more often responsible for requesting that preceptors observe their performance and complete the WBA [8]. The CC must consider all sources of assessment including WBA and make a decision about the trajectory of the resident toward competence.

Understanding the CC decision-making process is critical to ensuring that residency programs are using a rigorous program of assessment. A theoretical framework of CC decision-making was proposed by Chahine et al. [9], suggesting that the CC process was centred on one or a combination of three orientations: schema (well-structured, formulaic approach), constructivist (group constructs a shared understanding), and social influence (perspectives changes based on social pressures). Possible moderating factors including guidelines, timeframes, and leadership, might affect the CC discussions of resident assessment data leading to decisions about performance and feedback [9]. This model was expanded by Hauer et al. [10], who divided the CC process into four components, invoking different theories of group function. They proposed that social decision schema, functional theory (specifies functions necessary for effective decision-making), groupthink (desire for cohesion overrides careful consideration), and the Wisdom of Crowds (criteria for groups to make better decisions than individuals) could be combined to describe the process, adding Kane’s and Messick’s validity frameworks as mechanisms by which CC considered the consequences of their decisions [10]. These theoretical models of CC form a starting point, however, there are few in-vivo studies of CC functioning. One study of CC prior to implementation of CBME identified that most CC used a problem-identification approach to resident performance review while others were growth-oriented and used a developmental approach, providing feedback to every resident [11]. Early descriptions of CC data aggregation and decisions making have established best practices for meeting structure [12, 13]. More recently, Canadian groups have looked at the challenges faced by CC in data interpretation [14], potential roles of CC in residency assessment programs [8, 15] and the differential impact of CC on different residents [16]. Together, these studies illustrate the complexity of the CC process and highlight the importance of understanding factors that affect CC function in various contexts.

The models from Chahine [9] and Hauer [10] formed a starting point for understanding the CC decision-making process. They did not include direct study of CC, therefore, how closely the models reflected actual process was unknown. This qualitative study aimed to explore the methods used by two operational CC and their members, and to develop a model that explained the decision-making process.

## Methods

### Methodology

Case study methodology is ideally suited to explore and explain phenomena that are intrinsically linked with their contexts, including small group function and decision-making [17]. Our instrumental case study used in-depth examination of two cases to describe and understand the phenomenon of interest, with cases selected for their ability to illustrate the phenomenon and inclusion of two cases to gain a deeper appreciation of the topic [18]. The case study approach allowed us to disentangle anticipated complexities in the group relationships and interactions as we explored the factors affecting the decision process, understanding how competence and progress decisions were reached.

### Cases

The cases studied were the CC for two post-graduate training programs leading to certification with the Royal College of Physicians and Surgeons of Canada (RCPSC) at a single Canadian institution. The CC were purposively chosen as they were well-established in CBME, having implemented the new curriculum three years prior to the study (cases are detailed in Table 1). Importantly, the researchers had no direct relationship with the CC members or their trainees, minimizing potential conflict of interest. The bounds of the cases included all members of the CC during the time they spent reviewing files and in CC meetings, and the operational documents in place through the study period. All participants, including CC members and trainees, provided written informed consent for participation. The study was approved by the University of Calgary Conjoint Health Research Ethics Board and conducted according to relevant guidelines and regulations.

**Table 1:** Case descriptions

	**CC1**	**CC2**
Program duration	5 years	2 years
Number of trainees	4 per year	1–2 per year
Time since CC established	4 years pre-study	3 years pre-study
Experience of chair	2 years	3 years
Experience of CC	3 years	3 years
Composition of CC	5 male, 5 female members	3 male, 4 female members
	1 outside faculty representative	1 outside faculty representative
	Non-voting member as designated resident advocate	
CC Member experience	Two newer members with 1 year, remainder 3 years	All members 3 years
Meeting frequency	4 per year	4 per year
Sources of data considered	WBA, ITERs, in-training examinations, faculty advisor feedback, feedback on teaching sessions	WBA, in-training examinations, research output, feedback on teaching sessions, trainee self-assessment
Meeting format	Trainees presented by a designated reviewer on the committee; faculty advisor is arms-length from committee	Trainees presented by their faculty advisor who is a member of the committee

*RCPSC* Royal College of Physicians and Surgeons of Canada, *CC* Competence committee, *PD* Program director, *ITER* In-training evaluation report, *WBA* Workplace based assessment

### Data collection

#### CC Meetings and interviews

The study included sequential meeting observation and one-on-one interviews. Over six months, one researcher observed two meetings for each CC and conducted two or three semi-structured interviews within the two weeks following each meeting. All meetings and interviews were conducted using Zoom (Zoom Video Communications, Inc.). Meetings and interviews were recorded, transcribed verbatim, and de-identified prior to analysis.

The interview guide was established based on the research questions, pilot-tested using a think-aloud strategy [19], and refined. The interview guide was modified over time as informed by iterative analysis, to clarify points of interest identified in early observations and interviews. The number and choice of interviews was intentional. An open invitation was used early in the study. In subsequent rounds, members were invited for interviews if they were seen to have a key role in contributing to the decision process, including the member presenting trainee data or being an active participant in the discussions. The researchers met regularly to compare coding and observations. Data collection was halted after four meetings and 10 interviews, when no new ideas were emerging and the data contained ample depth to illustrate the variations within the themes [20].

#### Document review

The researchers generated field notes, memos, and annotations throughout the study; these were considered data and reviewed throughout. They included discipline-specific EPA (Entrustable Professional Activity) Guides, Requirements of Training, and Competencies documents from the RCPSC and program-specific CC Terms of Reference in the analysis.

### Data analysis

Within the case study framework, a constructivist grounded theory approach to data analysis was employed, recognizing that the researchers brought experience with residency training, supervision and progression decision-making, and knowledge of intended CC process to their analyses, which contributed to the theory that emerged from the data [21]. Data analysis began following the first meeting observed and proceeded iteratively throughout the study, using NVivo 12 (QSR International) for data management. Initial coding was done independently as open coding with line-by-line review of the transcript following the first CC meeting. The researchers met, reviewed the coding strategies, and prepared a working codebook; they re-coded initial transcripts and met again to revise and refine the coding strategy until satisfactory agreement and understanding was achieved. Subsequently, the researchers conducted independent and joint review of the transcripts, meeting frequently to ensure consistency of coding and that a mutual understanding of the data was emerging. Every 3 months, the researchers presented the preliminary data review to the research team. This allowed for the addition of their reflections and observations on the developing analysis. With repeated review of the transcripts, researchers identified emerging relationships and questions arising, which were captured in annotations and memos. Focused coding proceeded with categorizing and grouping of the initial codes to represent distinct themes. Particular attention was paid to similarities and differences between cases and specific decisions made during CC meetings.

Thematic coding was structured within an organizing framework, demonstrating relationships between codes and themes that could be integrated to explain the committees’ decision-making processes. The themes were refined by discussion with the research team and by referencing against the program documents. The preliminary findings and organizing framework were presented to participating CC members, allowing for member checking and additional reflection by the participants.

### Context

As qualitative researchers, we were aware of the impact of our backgrounds and intervention on the process that we observed and interrogated. C.C. was a program director in a postgraduate medical education specialty program and a graduate student in medical education. L.J.C. was a former program director and clinician educator with the RCPSC, involved in program evaluation. We considered our backgrounds and experience as contributing to the framework, guiding observation and interpretation of the data. Other members of the research team, post-graduate education researchers, knowledgeable with the intended goals and format of CBME, shared their insights and reflections throughout the study conception and analysis. The return of findings sessions helped to ensure that our assertions reflected the data but also integrated the medical education knowledge and personal perspectives of the participants.

## Results

### Data

The study included thorough examination of two CC, representing different residency training programs within the same Canadian institution. The transcripts from four CC meetings and 10 interviews, program-specific Terms of Reference documents, and RCPSC documents, together with the observations, memos and annotations generated by the study team formed the data for analysis. The observed meetings for CC1 each included the review of 12 trainees over 90 min and the CC2 meetings included 2–3 trainees over 30 min. The individual trainee discussions took an average of 6 min, 45 s (range 2:00–20:30); five discussions for trainees assessed as weaker had an average duration of 13 min compared to the standard trainee discussion averaging 5 min. A total of 28 trainee presentations and status decisions were observed over the course of the study, with some trainees represented more than once. Interviews lasted 45 to 60 min and participants were 10 of a possible 17 CC members including both CC chairs and PDs. Those not interviewed were three members who did not respond to invitations and four who did not attend the meetings observed.

### Coding

The initial coding structure included codes relating to the role and process of the committee, the data used by the committee and the group dynamics and discussion. Through repeated review and analysis of the data, the prevailing themes emerged as relating to process, data sufficiency, discussion triggers, interpretation, and mindset.

### Theme 1. CC role

#### Understanding of CBME

The joint understanding by members of the role of the CC and the guidelines that it should follow was fundamental to the decision-making process. For trainees who were doing well and more so for those who were struggling, questions about CBME arose. “I think there remains amongst the committee members some level of uncertainty with regards to by the book EPA counts that are needed to progress a trainee, versus overall gestalt based on their performance.” (Interview, F19). In multiple situations, the committees were unsure what criteria they should consider for trainee progression between stages. As one example, stated during CC1 meeting, “are the deficiencies we've noticed significant enough that you would want to hold them back out of Core? [Core is the third of four stages in the RCPSC CBME framework] [7] I'm not quite sure what the criteria is.” (CC1 meeting, F6) The implications of designating a trainee as progressing faster than expected and advancing early were discussed at length in one meeting, illustrating the difficulty in transitioning between time-based and competence-based training. This idea was clarified in interviews, with one member stating:

I think that's something that was not really thought about very carefully when EPAs were introduced - the question about the time frame. If you have someone that finishes everything within half the time, where does that put them, can they graduate sooner? Maybe not? (Interview, F13)

#### Committee evolution over time

Members of both CC identified that the functioning of their group had developed over time including a better understanding of CBME and how to assess trainees holistically in an efficient way. Their experience with the CC and CBME made the CC more comfortable in their roles and decision-making. This was described by one member as:

Traditionally, this committee reacted later than it ought to have in some cases. Now, the committee is more willing to make a decision [to identify a trainee as not progressing as expected] earlier on and trust the information and trust the process outside of this committee. (Interview, F5)

In addition, the experiences of the CC enabled them to adapt their structure to balance efficiency and completeness of discussion. Both cases used a template for presentation of data, that they felt ensured a thorough review of each trainee, “Using the progress checklist has really helped us be more holistic in speaking about the trainees.” (Interview, F12).

### Theme 2. Data sufficiency

#### Narrative vs. numbers

High quality assessment data was identified as essential to the process by the CC members. Many problems with data were identified, most commonly the need for illustrative and specific comments from observers. The CC members reported using the comments to help identify flags and verify that the entrustability score assigned accurately reflected the performance. This was especially important when there was discrepancy between the entrustability score and the narrative comment:

The WBA may have been scored as a 4 or 5 but the comments did not match that. Or the WBA was scored as 3/5 but the comments were “they were totally independent”. […] the Competence Committee is really only able to make global decisions when the data that they’re given is accurate and understandable. (Interview, F9)

This related directly to a second concern identified with WBA assessment scores: that some observers did not seem to understand the goal of the WBA or consider the context in which they were conducting the assessment. This was reflected in multiple comments, e.g. “The process has become a lot easier as preceptors become more acquainted with CBME, and have started to give more targeted feedback and understand the WBAs better” (Interview, F9), and, “many of them will have gotten low scores on that WBA but it's actually a misunderstanding of what the WBA is intended to measure”, (Interview, F2).

#### Trainee and CC approach to EPAs

The CC members were clear that the data contained in WBA assessment forms alone was insufficient to identify trainees who were not progressing as expected. The WBA data reflected assessments of select observations, most often requested by the trainee. This was described by one member as:

We struggled for the first few years that nobody had any of the lower scores, they were all 4 or 5. The trainees would wait and upon hearing “you did a good job on that [case]” they would ask, “can you fill in that WBA for me”. We knew that they were only targeting successful observations. (Interview, F2)

This led to a suspected over-representation of successful observations (four and five are high scores on WBA), raising concern that the data on file doesn’t show the global performance or learning over time. Members also questioned whether the absence of low-scoring WBAs should be considered as a point of concern, “I actually worry more about the ones that are consistently getting only fours or fives, that they're not putting themselves out there on the more challenging cases”, (Interview, F2).

The global assessment of WBA data by the committee was further complicated by trainee approach to WBA completion and the CC members’ consideration of WBA count. There were minimum numbers of WBA observations for each EPA required for progression through training, but RCPSC guidelines suggested that other factors should also be considered. Members of both CC agreed that simply achieving the minimum count of WBA was insufficient evidence to judge that a trainee had achieved competence:

It’s very tempting just to count the number of WBAs and say it’s good enough. But it’s always quite clear that the number of WBAs really doesn’t matter as much as how the person is actually performing. (Interview, F16)

Some trainees were recognized to be motivated to collect WBA observations and therefore achieved the minimum counts quickly, “One was able to fulfill [many more WBAs than their peers] within the same time frame of training. They were much more efficient in identifying what might apply as an [WBA observation for an] EPA and sending lots of requests”, (Interview, F13). Conversely, the failure to collect sufficient WBA observations could identify more complicated problems in trainees, “trainees that struggle seem to be “less good” at getting WBAs done. If they are barely meeting the minimums it’s usually a hint that there is a problem—but it may reflect problems with executive functioning” (Interview, F2).

In addition, not all aspects of medical practice were contained in the EPAs for a discipline. There were knowledge and performance metrics outside of the EPAs that merited consideration such as examination scores, “There have definitely been trainees who are progressing fine through EPAs but are having examination difficulties” (Interview, F5), and professionalism, “We had a trainee that was not progressing as expected; they had not completed the number of WBA in the expected amount of time, but more concerning was the identification of professionalism concerns, with not answering emails or completing documents or consults” (Interview, F12).

### Theme 3. Discussion triggers

In-depth discussions of trainee performance occurred for only five of the 28 trainee presentations in the meetings observed. The trigger for the discussion could be a concern identified by the reviewer or a question about the data presented to the committee,

the reviewer proposed progressing as expected […] but people heard comments coming through the objective feedback that made them think otherwise. Then we've had more in-depth discussion because it's been flagged by someone who's seen patterns arising (Interview, F8).

Most often, the discussion aimed to clarify the presence and source of a problem affecting a resident who was perceived to be struggling. There was also cognizance amongst CC members that there should not be too much weight placed on any one observation. The ensuing discussions tried to clarify how important the concern was, and whether it required action:

I worry sometimes that the comments are overvalued. We were talking about one trainee whose basic progression all looked fine, except for one comment. And there was a significant amount of discussion about that one comment. I think that’s OK, in the sense of the committee being informed. But I was concerned at the time, that we were going to put too much weight on this comment. (Interview, F5)

### Theme 4. Interpretation

#### Personal experience

While the goal of the CC to review documented assessments and make an objective, fact-based decision was stated in both CC Terms of Reference documents, the members found it difficult to separate their personal views of the trainee from the data. One member identified this as a form of bias,

Committee members do bring in their biases; I think that those biases are informed by a tacit judgment. You can't build a rubric that says, ‘is this person at their level or not’ […] when you don't have a comparator. (Interview, F2)

However, the concept was generally described as the members having personal, implicit impressions of a trainee’s competence, stated by one member as “If you've worked a lot with a trainee, they are now not just numbers on a page and words on feedback. You have a personalized vision of what that trainee’s performance is like.” (Interview, F10) CC members recognized that this knowledge could cloud their interpretation of the data in the file.

I think that there is the intangible impression that we get from trainees. […] The faculty presenting that trainee was perhaps too optimistic about their performance without the data to support it. In that instance the committee probably was correct [in voting down the motion, based on discussion of the data]. (Interview, F15)

#### Trainee context

Data interpretation was affected not only by the faculty’s personal experiences with the trainees and the assessment data, but also the trainee’s context and history. CC members acknowledged that trainees with a history of difficulties may have gotten a closer review of their file, “Once there is even a little flag or minor concern noted, that tends to roll forward with the trainee for at least a year or two”, (interview, F8). This could have led to a changed expectation for the trainee based on past performance, despite the stated CBME goal of norm-referenced assessment. Trainee context could also have affected the approach to progression decisions when there were suspected to be external factors affecting performance. For some trainees, their response to prior feedback made the CC hesitate in making a determination:

There was a significant conversation about how that decision [to not promote to Core] would impact the trainee’s mental health. In my view, that’s not the role of the committee. If the trainee is not progressing as expected, they deserve to know immediately so that they can have support. Delaying that because people have a big heart and are worried about the trainee, I don’t think it’s in the trainee’s interest to do that. (Interview, F5)

#### Effect of discussion

Despite their preformed impressions of trainees, CC members tried to listen to the data presented and participated in discussion to make decisions about trainees. They described that the discussion rarely changed their opinion about the trainees’ competence, but more often provided evidence to clarify their progress decisions. The members acknowledged that the program directors were often able to provide context or explanations that were beneficial in their interpretation. This was described by one member as:

It was through the discussions and what the Program Director and Assistant Program Director were adding from their perspective and with the EPAs that I was able to say, ‘I feel really comfortable with the idea that this trainee needs to be reviewed sooner.’ (Interview, F6)

In some circumstances, members reflected that although the group discussion changed the progress decision from the initial motion, there was little difference in their impression of the trainees’ competence.”No the change in progress status [from “progressing as expected”, to “progressing, minor concerns identified”] didn't change how I think about the trainee, it was really nuance to give the right message to the trainee […] not to change our general impression about the trainee,” (interview, F13).

### Theme 5. Mindset

A common theme recurring in the data was the mindset of the CC members and orientation of the committee. Some individual members did demonstrate a growth mindset in their contemplation of trainee progress. The trajectory of entrustability scores was considered as a way to visualize progress, “If the WBA scores look like a large percentage of them are in progress [scoring less than 4–5], then I'll look at the trend. If that shows they've been doing better over time, then great.” (Interview, F8) Another CC member described the process of feedback for learning as a key component of CBME and described that on one occasion, “the trainee said ‘I know that I'm not going to pass this’ and still wanted it evaluated. I appreciate that. […] filling out the WBA is not as important as sitting down and giving the trainee feedback on the performance”, (Interview, F15).

In contrast, some members consistently described and demonstrated a problem-identification orientation of the CC in trainee progress (they felt that they were there to identify and address problems, rather than to foster progression of all trainees). The difference in the time spent discussing and reviewing strong trainees compared to their peers was noticeable, along with the lack of developmental recommendations provided for them,

As far as the trainees who are doing well goes, there’s always the risk that those trainees fly by and are not necessarily either pushed or offered the level of constructive feedback that they should be getting. But whether the Competence Committee is the best place to identify that I’m not sure of. That seems to be more of an issue for the faculty advisors or Program Directors to assist with. (Interview, F9).

CC members described that they felt the role of the CC was to identify whether trainees were progressing as expected or not, and that further determination around goals and growth should take place in discussion with the program director or faculty advisor,

I see this committee as a bit of a screening for trouble and for identification of trainees who are having trouble and the like. […] this seems to be a lot more about the process of ensuring the residents are on track and doing okay and the work with the program directors and our education hub colleague is really where pushing new experiences is happening. (interview, F10)

This understanding of the roles of the CC and program director was in line with the CC1 terms of reference that described the role of the CC as advisory to the PD, while the CC2 terms of reference outlined the role of the CC without reference to the program director (Table 2).

**Table 2:** Role of the CC from program CC terms of reference

CC1	A regular review of trainees’ progress facilitates a developmental approach, supporting trainee learning over time. The CC should help the education team identify trainees who are not meeting their milestones and can suggest or mandate support and coaching for the trainee before the trainee gets too far off their trajectory. (CC1 terms of reference)
CC2	The mandate of the Competence Committee is to review and discuss learner portfolios in order to: • Advise and guide trainee learning and development; • Adjust a trainee’s training experiences to enhance learning opportunities; • Review assessments to determine a trainee’s achievement of each Entrustable Professional Activity (EPA); • Recommend learner status changes and progression of trainees through stages of training based on achievement of EPAs. (CC2 terms of reference)

### Committee process model

**Fig. 1:**
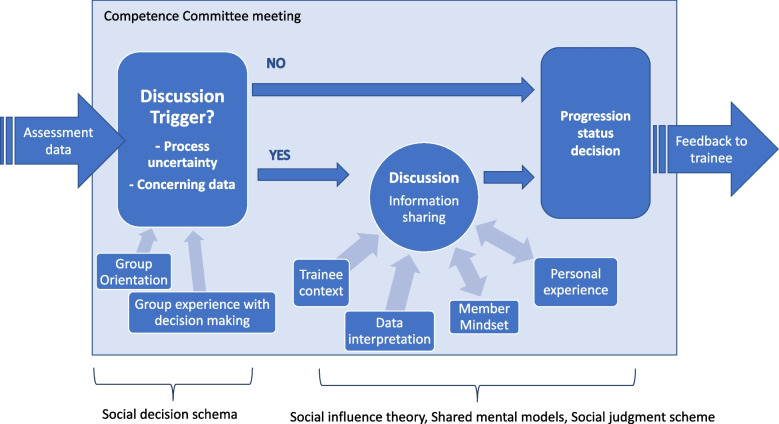
Model of the competence committee decision making process. Legend: The CC process follows primarily a social decision scheme that is moderated by the committee mindset, trainee context and experience of the group and individual members. When a question was raised about a trainee’s performance or the process to be followed, there was discussion invoking elements of social influence theory, shared mental models and social judgment scheme. The committee made a decision once there was sufficient understanding of the trainee’s progress

An understanding of the CC decision-making process emerged from our analysis and the resulting model is shown in Fig. 1. The CC process followed a social decision scheme that was moderated by the committee orientation, trainee context and experience of the group and individual members. In an organized and structured way, information was presented to the meeting in summary form by a CC member who had reviewed the data. The proposal and seconding of a motion could then proceed toward a decision one of two ways, based on whether the trainee was clearly meeting expectations or not. If the trainee was perceived to be doing well and the orientation of the committee was not developmental, no discussion ensued. The identification of uncertainty regarding trainee status was triggered by a single comment by a CC member who perceived a problem with the resident’s progress relating to the data presented or the process to be followed. In these situations, discussion ensued invoking elements of social influence theory, shared mental models and social judgment scheme. Members shared additional information to clarify the problem and come to a common understanding, including specific efforts to uncover unshared information held by the program directors or other members. The relative importance of the information shared by the PD and those who had more specific knowledge of CBME and of the trainee was impactful for members, exemplifying the social influence theory. This was explained by a member as “The program directors have mainly provided context and rarely thoughts as to what the decision ought to be. […] They’ve been an informative voice.” (Interview, F5).

The discussion to clarify the role and process of the CC refined the shared mental model of the task at hand including the effects of their decision on the trainee. This was explained “I think the group discussion was what really helped to solidify the correct decision for that trainee, taking all the different factors into consideration.” (Interview, F6). The role of personal experience with the trainee was reflective of social judgment theory; novel information presented was more likely to be accepted as true if congruent with the members’ pre-existing opinions. This was observed in one meeting where the discussion of whether to progress the trainee or hold them back due to concerns identified was summarized as, “This trainee, every review is always the same. They’re progressing as expected for them, perhaps not compared to all the others that we are assessing. For me, “progressing as expected for the candidate,” fits.” (CC2 meeting, F18). The depth of discussion was affected by the individual trainee’s context, prior experiences of the CC members and group experience with decision-making. The discussion provided experience to the CC members individually and as a group that could benefit understanding in future similar situations (represented by double arrows in the model). After the problem was understood to the satisfaction of the members, a decision was made about the trainee’s progress.

## Discussion

In this instrumental case study, we explored the decision-making of two CC representing programs of different size and length. In both cases, the CC demonstrated a problem-identification orientation, with meetings following a structured format for most trainees and invoking a more extensive discussion only when concerns were identified. The triggers for discussion consistently related to either CC members’ understanding of CBME and the CC role, or concerns with the data presented; ensuing conversations contained attempts to clarify the guidelines and understand the data in the context of the individual trainee and CC members’ experiences. Our findings fell within the theoretical description of group process outlined by Hauer and colleagues [10], and provided a case-based clarification of the relative importance of different decision-making processes for individual trainees. We presented a theoretical framework illustrating the CC decision-making process, as we understood it, following our theory-informed inquiry (Fig. 1).

The study of group process has led to many explanations of how small groups make decisions. The interplay of multiple theories in the theoretical framework proposed by Hauer et al., suggested that different aspects of committee function were explained by different theories [10]. Our study found that the CC followed a process that began within a social decision schema model and evolved to a discussion that invoked social influence theory, shared mental models, and social judgment scheme to clarify the points of contention. We identified that the CC decision-making was at risk of bias, primarily influenced by the group composition, the group orientation, individual members’ mindset, and their personal experiences with the trainees.

The desire to discuss trainees and identify areas for improvement, reflecting a growth mindset or belief that every individual has the potential to improve [22], was relevant to how frequently discussion triggers were raised. In an early description, CC were recognized to follow either a developmental or problem-identification model for resident review [11]; both CC in our study followed a problem-identification model. CC members in our study demonstrated growth mindset with respect to individual trainees, but they did not apply this in their meeting discussions. We observed a consistent difference in the time spent discussing and generating recommendations for perceived weaker residents, irrespective of the amount of WBA data available, as compared to high-achieving residents who were engaged with the WBA process and took initiative to collect large numbers of WBA assessments. This demonstrated a fixed mindset: that there was no need to try and help the high achievers as they would continue to succeed. While CC members acknowledged a risk with this approach, they felt that CC meetings were not the forum for discussing how to help trainees improve. Both CC delegated the responsibility of generating developmental feedback to the PD. These observations support the relationship between different types of residents and CC proposed by Rich and colleagues; that CC spent less time and provided less meaningful feedback to strong, engaged residents as compared to weaker residents and those less active in the process of seeking feedback and WBA assessments [16]. The discrepancy between expressed individual growth mindset and the group fixed orientation may be founded in the CC members’ uncertainty with respect to the role of the committee. It is recognized that mindset is not a fixed characteristic and could be changed by a motivated individual [22]. Analogously, it is plausible that a group could move toward a developmental orientation if its members agreed on the importance of the change.

Competence committees require a minimum quantity and quality of data to support their assessment of trainee progress, sufficient to support a high-stakes decision. In our study, members interviewed identified that the data contained in WBA assessments was insufficient for decision-making, particularly when the comments did not match the entrustment score. These findings are in line with previous reports on the use and interpretation of WBA for assessment. The use of entrustment scales has been demonstrated to be intuitive and reliable when used by trained observers [23, 24]. There was incremental benefit for committees in understanding the context of the trainee and to provide developmental feedback when the narrative feedback was detailed [25, 26]. Trainees identified that they appreciated the narrative feedback most for learning, but are more hesitant to request WBA when the feedback is not positive, limiting the utility of this tool by CC interested in gaining an overall view of the trainee performance [26, 27]. The concerns expressed regarding data sufficiency by CC members in our study may lead them to rely more heavily on their personal impressions of the trainees.

In agreement with previous publications [12, 28, 29], our study found that CC members formed a gestalt impression of trainee competence based on multiple assessments and considered their trajectory over a time, with their individual perspectives embedded in every discussion. This was apparent not as members sharing personal experiences and undocumented data, but rather their acceptance that, “it is impossible to completely dissociate your own personal perspective having worked with them because personal memories and interactions are always much stronger than looking at numbers,” (Interview, F8). The impact of this personal gestalt was amplified in CC2, a program with a small number of faculty and trainees, whose members recognized relying more on their impression of how the trainee was doing than on the data presented. There is value in expert opinion in the judgment of abilities and progress of trainees, this is the intention of the program of assessment’s inclusion of large quantities of feedback from multiple assessors over time. However, the CC decisions are at risk of visceral bias (judgment based on emotions), selection bias (reliance on partial non-representative information), or availability bias (preference to data that are more memorable) if the members rely too heavily on their prior experiences rather than the data [30].

The inclusion of members with diverse opinions and from varied contexts could increase sharing of novel information and perspectives, to ensure balanced discussion and minimize bias [31, 32]. The CC studied were both homogeneous, with a majority composition of clinical teaching faculty from within their specialties; the outside member on each CC represented a minority voice that may not have overcome the social decision structure. In establishing CC membership, programs should consider expanding the diversity of their committee with external members who have knowledge in CBME or assessment, thereby increasing the impact of their perspectives [33]. This is allowable but not required in the RCPSC guidelines, as, “Programs have the discretion to include additional members. Optional members might include an individual who is ‘external’ to the teaching faculty. This might be faculty or a program director from other residency programs at the university or from the same discipline at another university, other healthcare professionals, or a public member” [6].

### Limitations

This study was designed to examine the functioning of two CC in one institution and explored the process surrounding 28 trainee decisions to provide insight into the decision-making. The repetition of themes identified in the two different programs and the parallels with other CC studies increased the likelihood that our findings could apply in other settings. However, both programs studied were medical specialties whose collection and use of WBA data may differ from surgical specialties. The effect of personal experience-related bias seen in our small and medium-sized programs was perhaps more than in a larger program whose CC members have less direct involvement with every trainee. The CC members all recognized that their comfort with the work was increasing over time; it is possible that the reliance on personal impressions will be less when the CC have more confidence in the data they are provided with. While case study research is immersive and includes many sources of information, the trainee perspectives on CC were not included due to the initial research question focus on decision-making process; however, examining the downstream impact of those decisions would have added important insight.

Many of the CC members interviewed had experience in medical education, therefore their responses may have been based on their knowledge of the intended function of CC in addition to their experiences as CC members. This added to the complexity and richness of the findings, as the semi-structured interviews included thoughtful reflections on CBME as a system as well as the functioning of their CC.

## Conclusions

The conceptualization of CBME was of a learner-centred process designed to be individualized to each trainee’s needs and rate of learning [34]. We identified that the approach taken by CC and their members determined the benefits for trainees, and that this process was vulnerable to bias. The orientation of the committee, the sufficiency of data, and the personal experiences of CC members interacted in a complex decision-making process. Competence committees functioning with a problem-identification orientation of the CC resulted in high-achieving trainees received little valuable feedback from the CC. Faculty relied on their personal knowledge of the trainees to inform their decision-making, especially when the assessment data was insufficient in quantity or quality to support their decisions. These findings emphasize an ongoing need for faculty development in residency training programs post-implementation of CBME to mitigate potential sources of bias in CC functioning and to ensure that all trainees benefit.

### Supplementary Information

**Additional file 1. **

## Data Availability

The data that support the findings of this study are available on request from the corresponding author CC. The data are not publicly available due to them containing information that could compromise participant privacy.

## Abbreviations

CBME: Competency-Based medical education
CC: Competence committee
EPA: Entrustable professional activity
ITER: In-training evaluation report
PD: Program director
RCPSC: Royal College of physicians and surgeons of Canada
WBA: Workplace-based assessment
