# Supplementary material for: Competence committees decision-making; an interplay of data, group orientation, and intangible impressions
Source: BMC Med Educ. 2023 Oct 10;23:748. doi: 10.1186/s12909-023-04693-4 (PMC10565978; doi:10.1186/s12909-023-04693-4)
Supplement: Supplementary file 1 — Additional file 1. [file 12909_2023_4693_MOESM1_ESM.pdf]

Interview guide

Introduction

Thank you for participating in this interview. We appreciate you taking the time to share your views and impressions of the competence committee functioning. We are interested in the methods by which competence committees and their members make decisions about residents. This interview is being recorded so that it can be transcribed verbatim for qualitative analysis. Please do not identify residents or committee members by name during the interview; if this occurs, the transcriptionist will be instructed to remove and replace with "NAME".

It is possible that during review of the interview data, clarification might be needed and you could be contacted for a brief follow-up to verify that our interpretation aligns with what you intended to say.

Interview questions

1. How do you as a committee member make decisions about residents?
   Additional prompts:
   Prior to the meeting, which materials were most helpful in formulating your impression of the resident?
   What is the most challenging aspect of making decisions about whether the resident is at the appropriate level using this process?
   How do you weigh your own experience with the learner when you are working with the committee and assessment data?

2. Please tell me about how the competence committee makes decisions about residents.
   Additional prompts:
   What is done prior to the meeting, by whom, and what happens at the meeting?
   How is the process different for learners who are doing well vs. struggling?
   How is the process different for learners at transition points?
   How does the committee come to an agreement if there are strong differences of opinion?
   What would make the competence committee process easier?
   How has the use of virtual meeting technology affected your process?[1]

3. How does the committee use the resident assessments to inform their decisions?
   Additional prompts:
   What happens if the numeric score doesn't match the comments?
   How do you interpret narrative comments? Ambiguous or non-specific comments? Divergent comments?

4. Describe the group dynamics of your committee
   Additional prompts:

[1] Question added due to the COVID-19 pandemic changing meeting structure

Tell me about the power dynamics among the group?

Is there a dominant voice at the committee? IF yes – Why is that voice dominant? How does that voice influence other members' decision making?

What is the influence of the opinions of others and the discussion at the committee on your initial impression of the resident being reviewed?

When members of the committee disagree about the resident, what are the reasons?

How does the varied experiences of the committee members with residents in general and with the individuals being assessed contribute to the discussion?

5. How do you feel about the ability of the CC to assess resident ability and progression?

    Additional prompts:

    How would you describe the main role of your committee? How does this role differ between residents who excel and those who struggle?

    How does the CC identify residents in difficulty?

6. How does the CC consider the context requirements of the EPAs?[2]

7. How does the CC address the number of in progress assessments as compared to a resident who has only successful EPA observations?

    Does the CC show a growth mindset?

8. What would it take to hold someone back?[3]

    Would you consider rating a trainee as "progressing faster than expected"?

9. How does the CC decision get communicated back to the trainee?

    Is it different for those doing well vs those who are struggling?

10. Does the EPA count and progress through the CBD structure reflect true differences between trainees?[4]

---

[2] Questions 6 and 7 were added after 2nd committee meeting, for 2nd round of interviews

[3] Questions 8 and 9 were added after 3rd committee meeting, for 3rd round of interviews

[4] Question 10 was added after 3rd round of interviews
